# Motion Capture Data Analysis in the Instantaneous Frequency-Domain Using Hilbert-Huang Transform

**DOI:** 10.3390/s20226534

**Published:** 2020-11-16

**Authors:** Ran Dong, Dongsheng Cai, Soichiro Ikuno

**Affiliations:** 1 School of Computer Science, Tokyo University of Technology, Tokyo 192-0982, Japan; ikuno@stf.teu.ac.jp; 2 Faculty of Engineering, Information and Systems, University of Tsukuba, Ibaraki 305-8577, Japan; cai@cs.tsukuba.ac.jp

**Keywords:** motion capture data, motion analysis, motion primitive, feature extraction, Hilbert-Huang transform, empirical mode decomposition, Hilbert spectral analysis

## Abstract

Motion capture data are widely used in different research fields such as medical, entertainment, and industry. However, most motion researches using motion capture data are carried out in the time-domain. To understand human motion complexities, it is necessary to analyze motion data in the frequency-domain. In this paper, to analyze human motions, we present a framework to transform motions into the instantaneous frequency-domain using the Hilbert-Huang transform (HHT). The empirical mode decomposition (EMD) that is a part of HHT decomposes nonstationary and nonlinear signals captured from the real-world experiments into pseudo monochromatic signals, so-called intrinsic mode function (IMF). Our research reveals that the multivariate EMD can decompose complicated human motions into a finite number of nonlinear modes (IMFs) corresponding to distinct motion primitives. Analyzing these decomposed motions in Hilbert spectrum, motion characteristics can be extracted and visualized in instantaneous frequency-domain. For example, we apply our framework to (1) a jump motion, (2) a foot-injured gait, and (3) a golf swing motion.

## 1. Introduction

A motion capture system records a finite number of marking positions in time near the important human joints and convert them into their joint angles approximately using a simple human skeletal system. There have been numerous researches on motion analysis based on motion capture data in the different research fields [1]. Kim et al. [2] presented an analysis system and algorithm for golf swing based on an inertial sensor for sports motion analysis. Hssayeni et al. [3] developed an analysis method for estimating Parkinsonian tremor using both gradient tree boosting ensemble model and long short-term memory (LSTM) deep learning model. However, most researches focus only on the space-time domain because it is difficult to analyze and visualize in the frequency-domain. Besides, motion primitives are essential for deploying human activities [4], because intricated human movements are generated by combining and sequencing these motion primitives [5]. It is significant for motion analysis to decompose human motions into their primitives in the instantaneous frequency-domain.

Using Hilbert transform (HT), a real nonlinear monochromatic signal can be mapped into its imaginary part, and its instantaneous frequency and amplitude can be obtained [6]. However, because most real-world data are not monochromatic, they are not suitable for the HT. Huang et al. [7] proposed an empirical mode decomposition (EMD) in 1998, which can decompose a chromatic signal into several pseudo monochromatic signals, called intrinsic mode function (IMF), and a residual called trend. The pseudo monochromatic signals are used to satisfy the Hibert transform assumption A(t)cos(ω(t)t) and empirically obtained based on the definition of IMF. Decomposed IMFs can be transformed into an instantaneous frequency-domain using HT. This whole process is called Hilbert-Huang transform (HHT). Using HHT, human motion capture data can be analyzed in an instantaneous frequency-domain. There are also numerous researches on motion analysis in frequency-domain using Fourier transform (FT) or Wavelet transform (WT). Caramia et al. [8] studied the detection accuracy on different speeds of walk motions using WT. Kalampratsidou et al. [9] proposed methods to evaluate the self-emerging cohesiveness of ballet dances. This research use FT to convert motion capture data from the time-domain into the frequency-domain. Comparing with both FT and WT that transfer the signals into a series of linear sine and cosine waves, HHT decomposes the signal into a finite number of nonlinear IMFs and a trend (residual). In addition, bivariate EMD (BEMD), trivariate EMD (TEMD), multivariate EMD(MEMD) and noise-assisted Multivariate EMD(NA-MEMD) are also developed recently [10,11,12,13]. As a result, Hilbert spectral analysis (HSA) can be applied widely to various applications recently. Thus, for nonlinear, nonstationary, and multi-channel signals like motion capture data, HHT can be a powerful tool to analyze the nonlinear behavior of human motions.

A framework for motion analysis and dance editing using HHT has been proposed by Dong et al. [14,15]. However, these previous researches only propose that HHT can decompose dance motions into several IMFs based on a beat tracking algorithm [14,15]. In this paper, HHT is applied to a broader range of motions, and a framework for the human movement to extract motion features are proposed, and their decomposed motion dynamics for each IMFs are discussed. Using motion data published in the Carnegie Mellon University Motion Capture Database [16], we analyzed, for examples, (1) jump motion, (2) a gait of a foot-injured subject, and (3) a golf swing motion in the instantaneous frequency domain. Our results show that complicated motions can be decomposed into several distinct motion primitives such as jump motion, a gait of a foot-injured subject, and a golf swing motion in the instantaneous frequency-domain using our proposed framework. The characteristics of these decomposed motions can be analyzed and compared in detail focusing on these motion primitives.

## 2. Materials and Methods

### 2.1. Hilbert-Huang Transform and Empirical Mode Decomposition

The key idea of Hilbert-Huang transform (HHT) is that using empirical mode decomposition (EMD) to decompose a real-world signal into pseudo monochromatic waves, so-called intrinsic mode functions (IMFs). After decomposing a signal into several IMFs, their instantaneous frequencies and amplitudes can be calculated using Hilbert transform (HT). In this section, some HHT formulations are introduced. For the details, please see [17].

#### 2.1.1. Analytical Signal and Hilbert Transform

The analytical signal is a signal analysis theory widely used in the signal processing. The analytical signal is defined as $z\left(t\right)=z_{r}\left(t\right)+iz_{i}\left(t\right)$, where $z_{r}\left(t\right)$ and $z_{i}\left(t\right)$ denotes the real and imaginary part. Therefore, the instantaneous amplitude *A* and the instantaneous frequency ω can be obtained from the analytical signal as follows [6]:

(1) $$A\left(t\right)=\sqrt{{z_{r}}^{2}\left(t\right)+{z_{i}}^{2}\left(t\right)}$$

(2) $$\omega \left(t\right)=\frac{d}{dt}\arctan\frac{z_{i}\left(t\right)}{z_{r}\left(t\right)}$$

However, signals observed in the real-world data only have the real parts of them. To obtain the imaginary part of an observed signal, Hilbert transform (HT) assumes that the observed signal is a monochromatic wave $z_{r}\left(t\right)=A\left(t\right)\cos\left(\omega \left(t\right)t\right)$ and transforms the real part of the observed signal into its imaginary parts [6]:

(3) $$z_{i}\left(t\right)=\frac{1}{\pi }PV\int_{-\infty }^{\infty }\frac{z_{r}\left(\tau \right)}{t-\tau }\;d\tau =\frac{1}{\pi t}*z_{r}\left(t\right)$$

Here, PV denotes the Cauchy principal value [6]. The real part $z_{r}\left(t\right)$ of the analytical signal is observed in the real-world.

#### 2.1.2. Intrinsic Mode Functions (IMFs) and Trend

Because HT assumes the observed signal is a monochromatic wave A(t)cos(ω(t)t) and the real-world signals are almost chromatic, HT cannot be applied to these signals directly. The EMD was proposed by Huang et al. [17]. It empirically decomposes the chromatic signal into several IMFs (pseudo monochromatic waves) and a residual so-called “trend.”

The observed signal x(t) from the real-world can be defined as follows [17]:

(4) $$x\left(t\right)=\sum_{i=1}^{n}c_{i}\left(t\right)+r\left(t\right)$$

$\left\{c_{i}\left(t\right)|i=1,\ldots .,n\right\}$ is the set of IMFs, and r(t) is the residual. The definition of intrinsic mode function is as follows [17]:

- The number of signal extrema and the number of zero crossings points are equal, or their difference is 1; and
- At any time, the mean value of the envelope formed by the maximum, and the envelope formed by the minimum is zero.

After real-world data are decomposed into several IMFs, their instantaneous frequency and instantaneous amplitude can be obtained by HT.

#### 2.1.3. One-Variable Empirical Mode Decomposition

Using the definition of intrinsic mode functions and trend, the algorithm of EMD for a one-variable signal is as follows [17]:

1. Calculate residual r(t) (Let r(t)=x(t) in the first iteration);
  (5) $$r\left(t\right)=\sum_{i=1}^{n}c_{i}\left(t\right)-x\left(t\right)$$
2. Initialize $c_{\mathrm{old}}\left(t\right)=r\left(t\right)$ and extract one IMF; and
  - Find maximum envelope u(t) and minimum envelope l(t) of c(t) using cubic spline functions
  - Obtain $c_{\mathrm{new}}\left(t\right)$ by subtracting the average envelope from $c_{\mathrm{old}}\left(t\right)$
    (6) $$c_{\mathrm{new}}\left(t\right)=c_{\mathrm{old}}\left(t\right)-\frac{u(t)+l(t)}{2}$$
  - Let $c_{\mathrm{old}}\left(t\right)=c_{\mathrm{new}}\left(t\right)$ and repeat step 2 until the convergence condition (0.2≤SD≤0.3) is satisfied, add $c_{\mathrm{new}}\left(t\right)$ into the IMF set
    (7) $$SD=\frac{{\left(c_{\mathrm{old}}\left(t\right)-c_{\mathrm{new}}\left(t\right)\right)}^{2}}{c_{\mathrm{old}}{\left(t\right)}^{2}}$$
3. Subtract $c_{\mathrm{new}}\left(t\right)$ from r(t) and repeat step 1 and 2 to expand x(t) to all IMFs $\sum_{i=1}^{n}c_{i}\left(t\right)$ and a residual r(t).

#### 2.1.4. Multivariate Empirical Mode Decomposition

The multivariate EMD (MEMD) is proposed for signals such as motion data collected by multiple sensors [10]. The MEMD uses the Hammersley sequence to create the *n* dimensional sphere and project multivariate signals onto the sphere. By finding maximum and minimum envelopes covering the *n* dimensional sphere, we obtain the average envelope and subtract from the original multivariate signals separately. Using this algorithm, we can decompose multivariate signals such as human motions into multivariate monochromatic waves. Here, MEMD Algorithm can be described as follows [10]:

1. Perform a n−1 dimensional sphere created by Hammersley sequence;
2. Prepare several *n* dimensional unit vectors *V* (64 in this paper);
3. Make the projections ${\left\{p^{\theta_{v}\left(t\right)}\right\}}_{v=1}^{V}$ of the input multivariate signals s(t) for all channels, root position, and all Euler angles of each joint from a hierarchical human skeleton in motion, based on the directional vector *V*;
4. Determine the maximum and minimum positions ${\left\{t_{i}^{\theta_{v}\left(t\right)}\right\}}_{v=1}^{V}$ from the projections ${\left\{p^{\theta_{v}\left(t\right)}\right\}}_{v=1}^{V}$;
5. Create multi-dimensional envelopes ${\left\{e^{\theta_{v}}\left(t\right)\right\}}_{v=1}^{V}$ from the multivariate signals $s(t_{i}^{\theta_{v}})$ using cubic spline functions;
6. Calculate the mean m(t) from the directional vectors of all channels;
  (8) $$m\left(t\right)=\frac{1}{V}\sum_{v=1}^{V}e^{\theta_{v}}\left(t\right)$$
7. Obtain c(t)=s(t)−m(t) and repeat the above procedure to s(t)−c(t), unless the convergence condition of c(t) is satisfied. Then add c(t) as a decomposed pseudo monochromatic motion into the IMF set $\sum_{i=1}^{n}c_{i}\left(t\right)$ for all channels; and
8. Repeat steps 1–7 until all IMFs are obtained.

Please note that the decomposition is empirical. The number of IMFs depends on the number of embedded nonlinear signals in the signals themselves and the convergence condition Equation (7). Thus, the number of IMFs is not fixed. Discrete Fourier and Wavelet transforms transform the data into linear waves. The number of transformed waves depends on the sampling frequency. For biometric data such as brain wave data, motion data etc., which are mainly composed of nonlinear signals, HHT can be a very powerful tool to analyze the data.

#### 2.1.5. Weighted Average Frequency Algorithm

Boashsah shows that smoothing is possible if the instantaneous frequency is changing slowly enough [18]. Thus, the instantaneous frequency of each IMF obtained by HT can be smoothed. Niu et al. [19] developed a weighted average frequency algorithm (WAFA) that performs a smoothing for each IMFs. The algorithm smooths out the instantaneous frequency in three parts by the specified window length, as shown in Table 1.

Here, k=1,2,…,N (*N* is the number of data), *n* indicates sampling data, *j* indicates the number of IMF, ω indicates the instantaneous frequency of the decomposed IMF, *A* indicates the instantaneous amplitude of the decomposed IMF, and *m* is the length of the window.

Our previous research shows that WAFA can also be applied to human motion data [14]. Thus, WAFA can be used to smooth out IMFs from motion capture data. When the instantaneous frequencies vary faster than their own frequency, the instantaneous frequencies with smaller amplitudes have larger errors. For those instantaneous frequencies that do not have physical meanings in human motions, WAFA can smooth out these nonphysical instantaneous frequencies. Therefore, we use WAFA to obtain average frequencies of motions in the instantaneous frequency-domain.

### 2.2. Proposed Motion Analysis Framework Using Hilbert-Huang Transform

**Figure 1:**
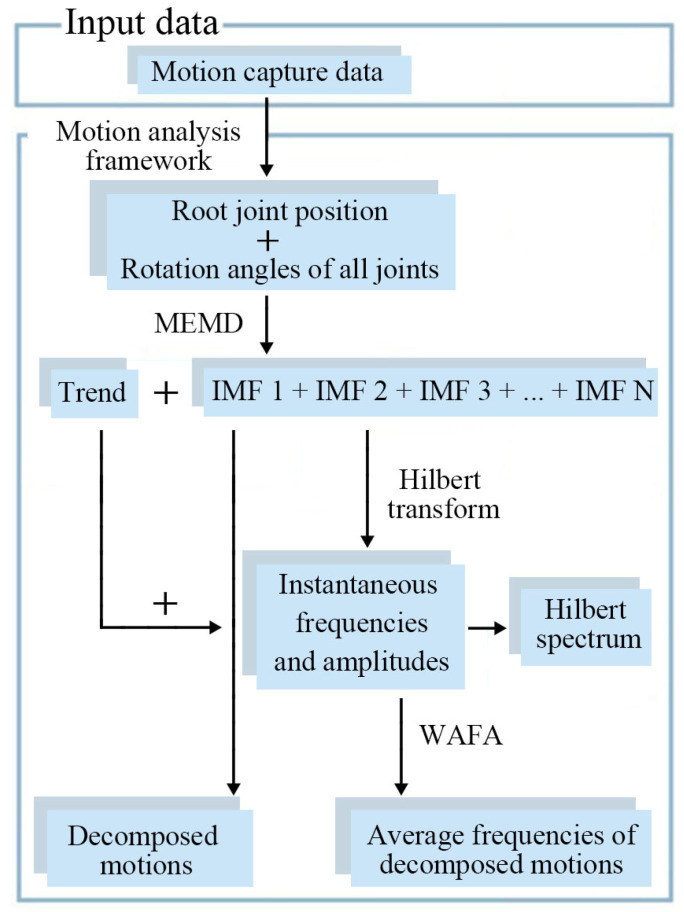
The proposed framework for motion analysis in the instantaneous frequency-domain using HHT.

This section proposes a framework to analyze human motions in the instantaneous frequency-domain. Figure 1 shows the proposed framework. It calculates the instantaneous frequency and instantaneous amplitude from motion-capture data using MEMD and Hilbert spectrum analysis.

We briefly explain the proposed framework as follows:

1. Prepare positions *X*, *Y*, *Z* of the root joint (hip), and three Euler angles $\theta_{x}$, $\theta_{y}$, $\theta_{z}$ of each joint obtained from the hierarchical skeleton. Here, the number of multivariate input channels is (root position) + (degrees of freedom) × (number of joints). For example, in the case of the motion data in Carnegie Mellon University Motion Capture Database, there are three positions of the root joint, three degrees of freedom of 43 joints, giving 132 channels;
2. Apply MEMD to all prepared data to obtain a set of IMFs and a trend for each multivariate input channel (N=132 for CMU database);
3. Output these IMFs and the trend as motion data, and confirm if the vibration components (decomposed motions) are completely separated;
4. Apply HT to each IMF to obtain the instantaneous frequencies and instantaneous amplitudes.
5. Apply WAFA to smooth out the instantaneous frequencies of each IMF to obtain the average frequency of each decomposed motion; and
6. Analyze decomposed motions with the HT spectrum and average frequencies (angular velocities of motion primitives) in the frequency-domain.

### 2.3. Input Data and Joint Angles $\theta_{x}$, $\theta_{y}$, $\theta_{z}$

**Figure 2:**
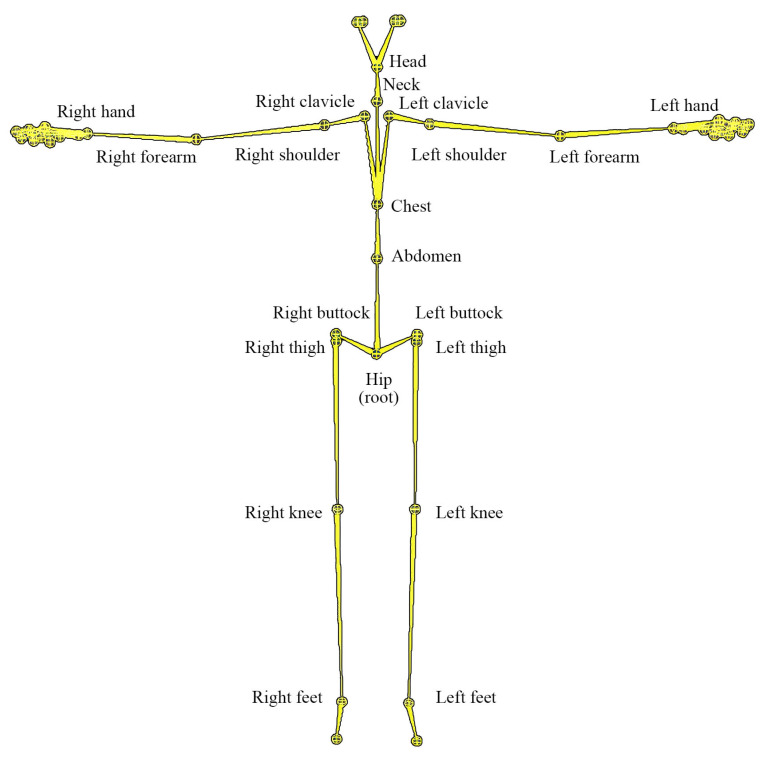
The skeleton hierarchy of CMU motion capture data.

In this paper, we use motion capture data obtained from the CMU motion capture database [16]. The sampling rate of motion data is 120 Hz. Figure 2 shows the skeleton hierarchy of motion data described as BVH (Biovision Hierarchy). Each motion data have 129 angles at 43 joints throughout the body and 3 positions of one root joint (hip).

**Figure 3:**
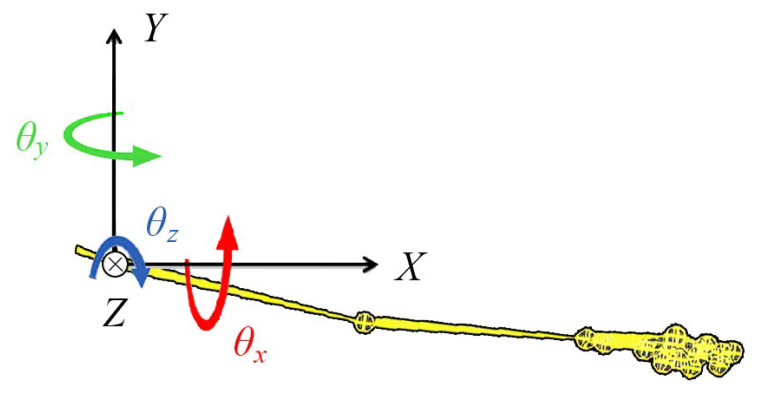
The skeleton hierarchy of CMU motion capture data.

Figure 2 shows the origin pose (T-pose: all joint angles $\theta_{x}$, $\theta_{y}$, $\theta_{z}$ are zero). Here, the skeleton model uses a hierarchical recording method in which the root origin is placed in the hip. For each joint, as shown in Figure 3, each joint of the body moves with 3 degrees of freedom $\theta_{x}$, $\theta_{y}$, $\theta_{z}$.

When people move their bodies, each joint moves by Euler angles: $\theta_{x}$, $\theta_{y}$, $\theta_{z}$. In HT spectrum analysis, it is desired to focus on the joints where the motions are concentrated. For example, a jump motion is mainly concentrated on the hip joint, and a golf swing motion is concentrated on the hand joints.

### 2.4. Decomposed Motions

Because we use the joint angles of a skeletal hierarchical model as the input data, each decomposed IMFs can be considered as the nonlinear oscillations generated from the root joint (hip) to the end joints (for examples, feet, hand, and head). The trend can be considered as the posture that varies in time. Because IMFs are nonlinear oscillations, it is necessary to add the trend to all IMFs to render the decomposed motion primitives.

## 3. Results

In this section, we apply our proposed framework to three different motions: (1) a jump, (2) a gait of foot injured subject, and (3) a golf motion. They are listed in Table 2. All these motions are composed of several motion primitives. Table 2 also shows the times and frequencies of the three motions. The Hilbert transform (HT) takes the second derivative of the original data. For Discrete HT, we take finite differences two times. Thus, the sampling rate reduces to one fourth [6]. The CMU motion data are sampled at 120 Hz. Those discrete HT transformed data are limited to 30 Hz. However, in our proposed motion analysis, 30 Hz is good enough because almost no significant joint motions are observed higher than 20 Hz.

These three motions use different joints to perform the motions. The jump, the gait of foot injured subject, and the golf swing motions mainly move the hip, the feet, and the right-hand joints, respectively. Thus, we show the Hilbert spectrum of main joints to analyze the decomposed motions.

### 3.1. Jump Motion Decomposition

**Figure 4:**
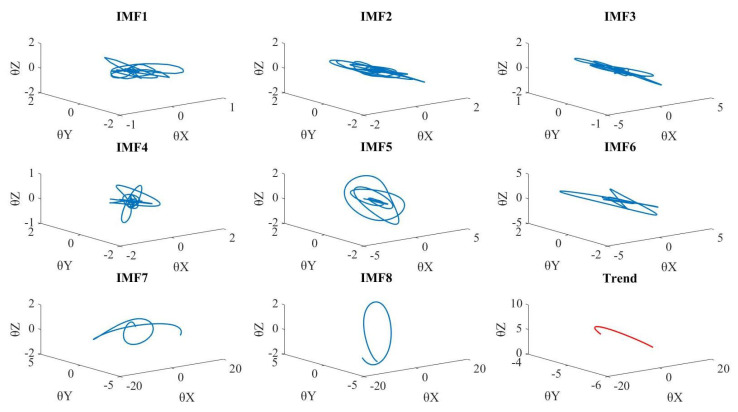
The hip joint’s decomposed motion $\theta_{x}$, $\theta_{y}$, $\theta_{z}$ using MEMD.

**Figure 5:**
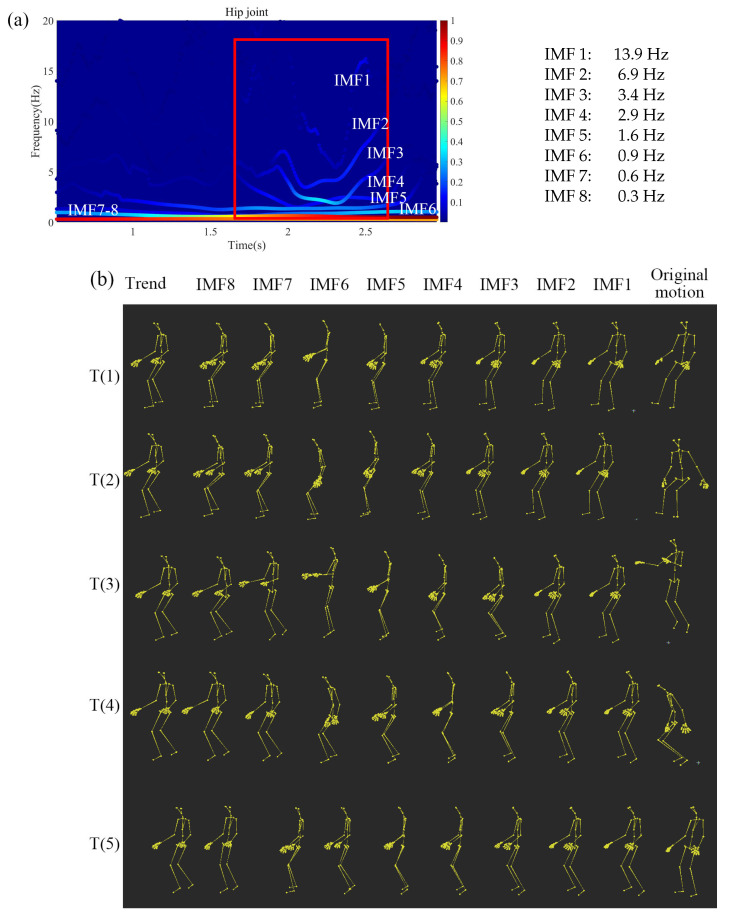
Decomposed jump motions using our proposed framework. (**a**) The Hilbert spectrum of the hip joint, and (**b**) each IMFs and the trend decomposed by MEMD. Here, T(1)–T(5) correspond to the time 0.5 s, 1.0 s, 1.6 s, 2.2 s, and 2.8 s.

Figure 4 shows the decomposed hip-joint motion plotted in 3 Eulerian angles $\theta_{x}$, $\theta_{y}$, $\theta_{z}$. As we can see from the figure, the motions have been decomposed from high frequency to low frequency by MEMD. We can also see the jump motion starts at 1.7 s and ends at 2.5 s, as shown in Figure 5a. Thus, comparing these decomposed motion data using IMFs, it is possible to analyze the motion features in the instantaneous frequency-domain.

Figure 5a shows the jump motion spectrum of each IMF decomposed by MEMD, the average frequencies of each IMF obtained by WAFA are listed on the right. Figure 5b shows the decomposed motion in the instantaneous frequency-domain. Because the jump motion mainly uses the hip joint to perform the movement, we focus on the hip motion. As we can see from Figure 5a, the jump motion can be divided into IMFs from high to low frequencies. The whole jump motion can be divided into two parts. One is the jump motion itself, starting at 1.7 s and ending at 2.5 s, which is composed of the IMF 1–5 corresponding to different motion primitives indicated by a red frame. Here, IMF 1–2, 3–4, 5–6, and 7–8, respectively, correspond to the (1) reactive motion hitting the ground, (2) the kicking motion, (3) the starting and ending motions, and (4) the remaining motions, including preliminary and crouching actions. Thus, as shown in Figure 5, the jump motion can be decomposed into different distinct motion primitives in the instantaneous frequency-domain.

Omkar et al. [20] performed short and long jump motion analysis, and their research shows both short jump and long jump have the reaction force decreasing from foot, knee, thigh hip to the neck joint when landing the ground [20]. Table 3 and Table 4 show our results and the instantaneous frequency ω and amplitude *A* of each decomposed IMF and the trend when landing the ground. It can be confirmed from Table 3 that the instantaneous amplitude, which indicates the wave intensity or the movement of the body, clearly decreases from the foot, knee, thigh hip to the neck joint. This coincides with previous research [20].

### 3.2. An Injured Gait Motion Decomposition

**Figure 6:**
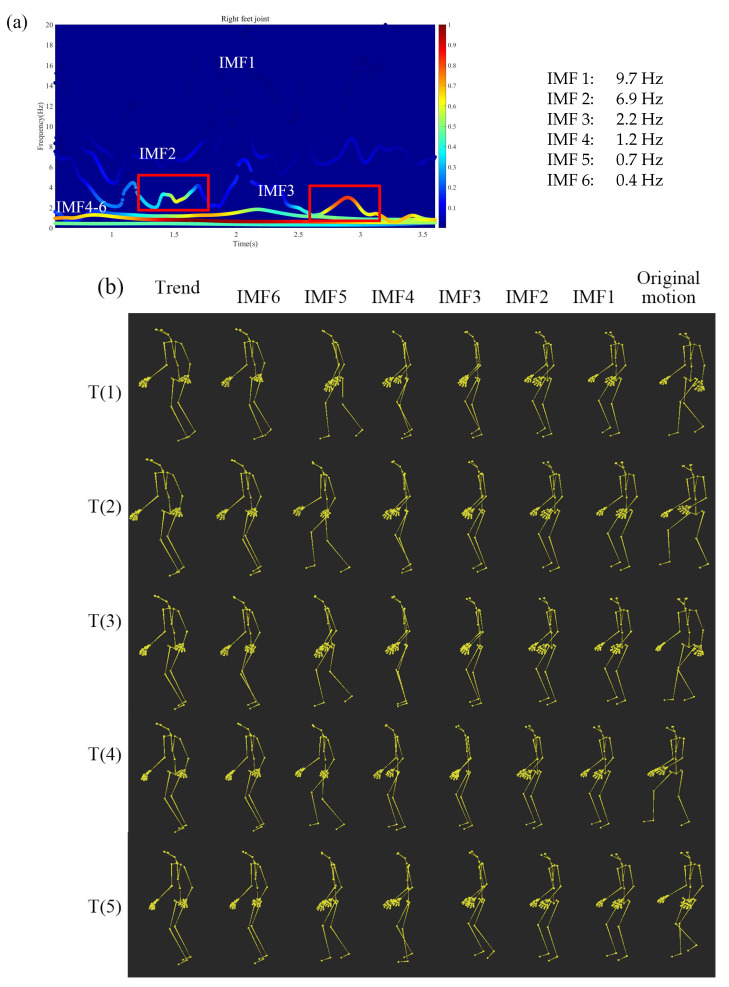
Decomposed gait motions of foot injured subject using our proposed framework. (**a**) The spectrum of the injured right foot joint. (**b**) Each of the IMFs and the trend motions decomposed by MEMD. Here, T(1)–T(5) correspond to the time 0.5 s, 1.2 s, 2.0 s, 2.8 s, and 3.5 s.

Figure 6a shows the Hilbert spectrum of a gait of a foot injured subject. We focus on the right injured foot joint to analyze the movement. As displayed in Figure 6a, the motion is decomposed into six IMFs in the instantaneous frequency-domain. Figure 6b shows the decomposed movements of each IMFs and the trend. As displayed in the figure, IMF 1, 2, 3, 4–6 are, respectively, (1) the noise motion from the motion capture data, (2) the reactive motions from the ground, (3) the characteristic injured foot motion compared with the normal motion, and (4) the low-frequency normal gait motion. The IMF 3 Hilbert spectrum has two motions that characterize the injured motions. The two motions are surrounded by the red square. Comparing and localizing the injured motion of IMF 3 with other decomposed gait motions, it is possible to analyze the walking pattern with injured foot.

Sorenson et al. [21] presented a method to reduce athletes’ injuries by calculating the two-dimensional and three-dimensional correlations between different joints. They discussed correlation coefficients *R*s with those of other researches. As shown in Table 5, to demonstrate our framework, we calculate the correlation coefficients *R*s between the injured right leg and left leg joints of each IMF, respectively. In the gait motion, the right and left legs move in turn. We expect the characteristic injured motion, i.e., IMF 3 of injured knee joints have less correlation between left and right in frequency. On the other hand, we expect the characteristic injured motion, i.e., IMF 3 of injured knee joints have a higher correlation between left and right in amplitude. This evidences that the injured left and right knee motion, respectively, synchronize and asynchronize in frequency and amplitude to support each other. This also evidence that the injured motions are localized and characterized in IMF 3.

### 3.3. A Golf Swing Motion Decomposition

**Figure 7:**
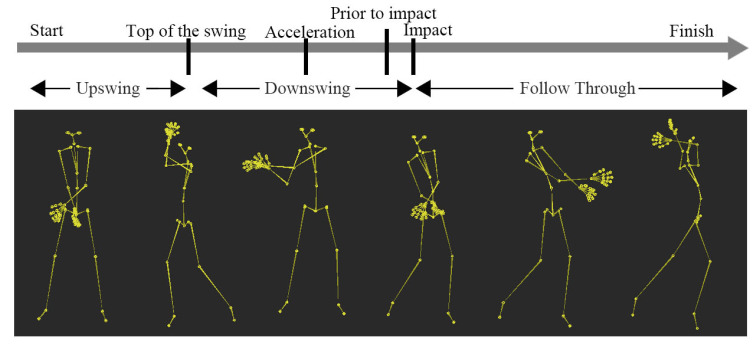
Selected events of a golf swing motion [23].

The golf swing motions have been studied in different areas due to its complexity. Many researches on the golf swing have been conducted in different research fields, such as engineering and sport science. Nam et al. [22] present an algorithm based on two sensors to analyze and track the golf swing. Chu et al. [23] propose several coaching ideas to increase ball velocity. Their research divides the golf swing motion into three parts: (1) upswing, (2) downswing, and (3) follow-through based on the time series to analyze golf swing motions, separately. Their studies also label essential motion points that can distinguish aoverlapped motion primitives: (1)start, (2)top of the swing, (3) acceleration, (4) prior to impact, (5) impact, and (6) finish, as indicated in Figure 7 [23].

**Figure 8:**
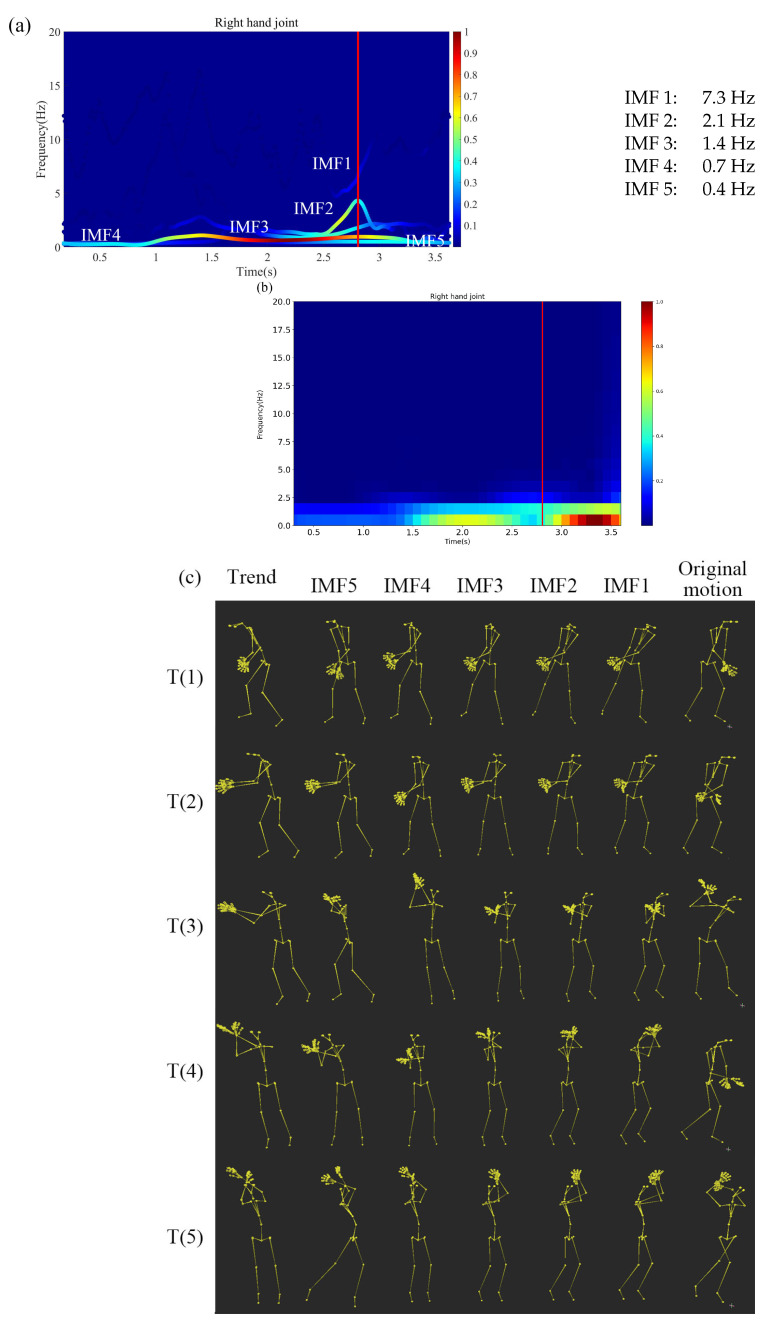
Decomposed golf swing motions using our proposed framework. (**a**) The spectrum of right hand joint. (**b**) STFT (Short Time Fourier Transform) spectrum of the right hand joint. (**c**) Each of the IMFs and the trend motions decomposed by MEMD. Here, T(1)–T(5) correspond to the time 0.3 s, 1.1 s, 1.9 s, 2.7 s, and 3.5 s.

Based on the previous golf swing researches, our results show that higher frequency IMFs correspond to “impact”, “prior to impact”, “acceleration”, and lower frequency IMFs correspond to “up- and down-swings” and “follow-through.” Figure 8a shows the Hilbert spectrum of decomposed golf swing motion, the red line (about 2.7 s) indicates the impact point in Figure 8a. Comparing with Figure 8a,b shows short-time Fourier transform (STFT) spectrum of the same golf swing motion, where the window length is 120 frames per 1 second and, the stride is 10 frames per 0.1 s. It is apparent that Hilbert power spectrum using HHT are much clearer than those using STFT. Figure 8c shows the decomposed motions. As we can see from the spectrum, IMF 1, 2, 3, 4, and 5 are, respectively, correspond to: (1) the “impact” that is the highest frequency motion, (2) “prior to impact” that is gripping and right elbow motion prepare for the impact, (3) “acceleration” that accelerates the swing, (4) “up- and down-swings” corresponding to the “Start,” and (5) “follow-through” corresponding to the “Finish.” Using our proposed framework, the complicated golf swing can be decomposed into (1) to (5) motion primitives that can be used to analyze the human mechanics and be used for training purposes.

As we discussed before, the golf swing motion can be decomposed into several distinct motion primitives using our framework. Thus, we can also calculate the root mean squared errors of each frame for each decomposed IMF and trend like previous researches [24,25] to give a quantitative evaluation. Because the golf motions are concentrated on a human body’s arms, we selected shoulder, forearm, and hand joints to calculate root mean squared error for each decomposed IMF and trend as shown in Table 6. Thus, we can analyze complicated human motions as the decomposed motions based on IMFs corresponding to their motion primitives, quantitatively.

We can also analyze golf motion based on the previous research in the instantaneous frequency-domain. In the previous paper, Chu et al. [23] used “Top”, “Acceleration”, “Last 40 ms”, and “Impact” event to evaluate and analyze the golf swing motion for each joint. For example, in our research, we calculate the right hand joint instantaneous frequency and instantaneous amplitude of each IMF shown in Table 7. The previous study [23] show that from “Top” to “Impact”, the wrist hinge rotational velocity increases. Our result also indicates that increases in IMF 1–3 exist. They correspond to “impact”, the “prior to impact”, and the “acceleration”, respectively.

## 4. Discussion

This study presents our framework to decompose motion capture data into the distinct motion primitives in the instantaneous frequency-domain using HHT. We apply our method to three different motions: (1) a jump, (2) a gait of foot injured subject, and (3) a golf swing.

First, we use a jump motion as an example to show the decomposition results. The jump motions are also considered as a complicated motion. Omkar et al. [20] integrated a low-cost method for motion analysis using a three-axis accelerometer, a three-axis magnetometer, and a microcontroller, which are very accurate and easy to use in the short and long jump motion analysis. This study reveals that jump landing impact crates different accelerations from the foot to the neck [20]. Our proposed framework can also decompose a jump motion into two different accelerations generated by the jump landing impact in the high-frequency-domain (IMF 1–2), as shown in Figure 4. Other decomposed motions (IMF 3–8) correspond to each motion primitives based on the human body’s hierarchical structure. Using our method, we can analyze different types of jump motions in the instantaneous frequency-domain.

Second, we apply a gait of a foot injured subject to our method. There are some researches on injury motions. Sorenson et al. [21] discuss two and three-dimensional relationships between the knee and hip joints to reduce athletes’ injury. Our research evidences the distinct injured motion primitives that are localized and decomposed in the instantaneous frequency-domain. The decomposed distinct injured motion primitives can help researchers to understand the injured motion patterns for rehabilitation purposes.

Finally, we decompose a golf swing motion into five known distinct motion primitives in the instantaneous frequency-domain using our framework. Our results coincide with the previous research [23] and can be used for training purposes.

Comparing Figure 8a,b, HHT clearly decomposes 5 golf motions that are (1) the “impact”, (2) “prior to impact”, (3) “acceleration”, (4) “up- and down-swings”, and (5) “follow-through”. They correspond to IMF 1 to 5, respectively, while STFT spectrum in Figure 8b can barely distinguish 2 modes that possibly correspond to IMF 3 and 5 in (a). The complicated and important motions near the impact (t~3 s, red bar) are almost invisible in Figure 8b. In addition, as shown in Figure 8a, HHT clearly decompose 7Hz impact motion (IMF 1) on the red line. The EMD used in HHT is only empirical. It is difficult to evaluate the correctness and accuracy of these decomposed motions mathematically. However, at least, they are consistent and STFT cannot decompose them clearly. We believe that HHT has certain advantages to analyze the nonstationary and nonlinear signals over Fourier transform, wavelet transform etc.

Unfortunately, there is no mathematical proof that evidence the chromatic signal can always be correctly decomposed into monochromatic signals using EMD because the decomposition algorithm is only “empirical”. Thus, mode mixing may occur during the decompositions that generate singular IMFs [26]. As a result, the average frequencies of each IMF cannot be obtained correctly. To resolve this problem, basis pursuit denoising (BPD) [27] can be used to minimize the mode mixing. The BPD as a preprocessing purpose can reduce singular IMFs that cause mode mixing and IMF singularities.

Previous research [14,15] only shows that the motion data can be decomposed into IMFs with a trend (posture). In the present paper, complex human motions are decomposed into several IMFs corresponding to the distinct motion primitives in the instantaneous frequency-domain. We show these decomposed IMF motions are distinct motion primitives that have different physical meanings. Our framework can be a useful and powerful tool to analyze complicated human motions.

## 5. Conclusions

Traditional data analyses are based on linear methods. However, in most real-world experiments, whether natural or artificial, the data are likely to be nonlinear and noisy. To resolve this problem, HHT uses EMD to decompose a real-world signal into a set of IMFs and a residual (trend). The IMFs can decompose the chromatic signal into pseudo monochromatic signals. Thus, we can apply the Hilbert transform to each IMFs to obtain both the instantaneous frequency and amplitude of the original signal. The HHT has been utilized in a wide range of researches. However, few researches focus on motion analysis using HHT, which are nonstationary and nonlinear, and obtained from real-world experiments. In this paper, we propose a framework for human motion analysis using HHT. To evidence the significance of our framework, we use three motions, for example, to show our proposed method can decompose the complicated original motion into several distinct motion primitives. This is impossible using STFT. We apply our framework to three different motions: (i) a jump motion; (ii) a gait of foot injured subject; and (iii) a golf swing motion. Three different decomposed motions show that our framework can decompose complicated human motions into their distinct motion primitives in the instantaneous frequency-domain. The Hilbert spectrum and decomposed motions show that the decomposed motions correspond to the distinct motion primitives that are localized and physically different. Thus, the targeted characteristic motions can be focused and analyzed. Our framework can be a significant tool for human motion analysis in the future.

## Figures and Tables

**Table 1:** Weighted Average Frequency Algorithm (WAFA) [19].

*k*	Average Frequency for Each *k*
$1\leq k\leq \frac{m-1}{2}$	$\bar{\omega_{j}}\left(k\right)=\frac{\sum_{n=1}^{k+\frac{m-1}{2}}\omega_{j}\left(n\right)A_{j}\left(n\right)}{\sum_{n=1}^{k+\frac{m-1}{2}}A_{j}\left(n\right)}$
$\frac{m+1}{2}\leq k\leq \frac{2N-1-m}{2}$	$\bar{\omega_{j}}\left(k\right)=\frac{\sum_{n=k-\frac{m-1}{2}}^{k+\frac{m-1}{2}}\omega_{j}\left(n\right)A_{j}\left(n\right)}{\sum_{n=k-\frac{m-1}{2}}^{k+\frac{m-1}{2}}A_{j}\left(n\right)}$
$\frac{2N+1-m}{2}\leq k\leq N$	$\bar{\omega_{j}}\left(k\right)=\frac{\sum_{n=k-\frac{m-1}{2}}^{N}\omega_{j}\left(n\right)A_{j}\left(n\right)}{\sum_{n=k-\frac{m-1}{2}}^{N}A_{j}\left(n\right)}$

**Table 2:** Three different motion data for decompositions.

Motion Capture Data	Jump	Gait of Foot Injured Subject	Golf Swing
Time (s)	3.3	3.7	3.8
Maximum frequency (Hz)	30	30	30
Minimum frequency (Hz)	0.30	0.27	0.26

**Table 3:** The instantaneous frequency (Hz) of each decomposed IMF and its trend at the landing impact.

IMF	Foot	Knee	Thigh	Hip	Neck
1	11.8	11.6	12.3	22.08	5.93
2	6.58	6.21	6.71	6.95	5.96
3	4.7	3.66	2.33	3.99	4.01
4	1.7	2.39	1.65	2.42	1.88
5	1.53	1.42	1.46	1.46	1.02
6	1.04	1.09	0.92	0.95	0.88
7	0.57	0.58	0.54	0.53	0.54
8	0.33	0.3	0.28	0.3	0.3

**Table 4:** The instantaneous amplitude (deg) of each decomposed IMF and its trend at the landing impact.

IMF	Foot	Knee	Thigh	Hip	Neck
1	2.86	0.37	0.86	1.21	0.31
2	3.08	3.2	2.24	1.7	0.33
3	4.73	5.91	1.78	1.98	0.7
4	4.34	3.43	4.4	1.78	1.04
5	10.03	11.07	6.6	2.95	0.49
6	18.4	22.61	12.78	3.81	2.05
7	20.26	16.35	15.3	11.5	2.76
8	7.06	15.62	16.84	8.8	3.06

**Table 5:** The correlation coefficients *R*s of each decomposed IMF instantaneous frequency and amplitude between hip and leg joins (thigh, knee, foot) of whole gain motion.

	Instantaneous Frequency			Instantaneous Amplitude		
IMF	Foot	Knee	Thigh	Foot	Knee	Thigh
1	0.89	0.86	0.72	0.8	0.68	0.93
2	0.72	0.84	0.64	0.27	0.39	0.91
3	0.48	0.63	0.61	0.67	0.73	0.8
4	0.52	0.35	0.67	0.25	−0.13	0.94
5	0.65	0.66	0.27	0.02	−0.18	0.95
6	0.9	0.85	0.98	0.3	0.26	0.93

**Table 6:** Root mean squared errors of each frame for the decomposed IMFs and the trends of golf swing.

Error (Deg) RMS (Mean) ± STD						
	**Right**			**Left**		
No.	Shoulder	Forearm	Hand	Shoulder	Forearm	Hand
IMF 1	0.13 ± 0.22	0.04 ± 0.08	0.09 ± 0.2	0.1 ± 0.17	0.03 ± 0.07	0.09 ± 0.15
IMF 2	1.1 ± 1.51	0.48 ± 0.84	0.84 ± 1.4	0.61 ± 0.71	0.2 ± 0.29	0.89 ± 1.36
IMF 3	2.51 ± 2.97	0.66 ± 0.86	1.88 ± 2.26	2.34 ± 2.3	0.71 ± 0.8	1.77 ± 2.22
IMF 4	4.24 ± 2.17	3.72 ± 2	3.77 ± 2.41	5.21 ± 2.21	1.61 ± 1.14	2.54 ± 1.42
IMF 5	6.92 ± 2.47	6.1 ± 2.67	2.07 ± 1.18	9.05 ± 2.02	2.75 ± 1.6	3.46 ± 1.47
Trend	29.5 ± 2.95	19.69 ± 5.17	9.43 ± 5.79	36.29 ± 1.74	11.56 ± 4.6	6.95 ± 2.28

**Table 7:** Right hand joint rotational instantaneous frequency (Hz) of golf swing.

IMF	Top	Acceleration	Last 40 ms	Impact
1	3.06	4.95	6.74	7.92
2	1.43	1.75	4.54	3.76
3	0.95	1.14	1.76	1.96
4	0.65	0.85	0.93	0.94
5	0.45	0.48	0.51	0.51

## Abbreviations

The following abbreviations are used in this manuscript:

HHT	Hilbert-Huang transform
HT	Hilbert transform
FT	Fourier transform
WT	Wavelet transform
EMD	empirical mode decomposition
IMF	intrinsic mode function
MEMD	multivariate empirical mode decomposition
CMU	Carnegie Mellon University
